# Acute Anterior Uveitis as a Risk Factor of Ankylosing Spondylitis—A National Population-Based Study

**DOI:** 10.3390/ijerph14010107

**Published:** 2017-01-23

**Authors:** Ju-Chuan Yen, Chia-An Hsu, Sheng-Huang Hsiao, Min-Huei Hsu

**Affiliations:** 1 Graduate Institute of Biomedical Informatics, College of Medical Science and Technology, Taipei Medical University, Taipei 11031, Taiwan; m701061@gmail.com; 2 Department of Ophthalmology, Ren-Ai Branch, Taipei City Hospital, Taipei 10629, Taiwan; 3 School of Medicine, National Yang-Ming University, Taipei 11221, Taiwan; johnhsu129@gmail.com; 4 Department of Neurosurgery, Ren-Ai Branch, Taipei City Hospital, Taipei 10629, Taiwan; 5 Department of Psychology, National Chengchi University, Taipei 11605, Taiwan; 6 Ministry of Health and Welfare, Taipei 11558, Taiwan

**Keywords:** acute anterior uveitis (AAU), ankylosing spondylitis (AS), National Health Insurance Research Database (NHIRD)

## Abstract

Introduction: In clinical settings, acute anterior uveitis (AAU) could be the first presentation of ankylosing spondylitis (AS). Based on this hypothesis, we investigate whether AAU is a risk factor in developing AS later by using National Health Insurance Research Database (NHIRD) in Taiwan. Materials and Methods: This cohort comparison study used longitudinal Taiwanese NHIRD to probe the relative risk odds of AAU for AS development, and consisted of all patients diagnosed with AAU (n = 5621) (ICD-9-CM codes 364.00). The relative risks of AS between AAU patients and controls were compared by estimating the crude hazard ratio with logistic regression. Kaplan–Meier analysis was used to calculate the cumulative incidence rates of developing AS, and a log-rank test was used to analyze the differences between the survival curves. Separate Cox proportional hazard regressions were performed to compute the AS-free rate after adjusting for possible confounding factors such as age and sex. Results: The crude hazard ratio was 2.667 for the AAU group, and the adjusted hazard ratio was 2.705 for the AAU group. The observation time of the AS-free group was shorter for AAU patients compared with the control group (1507 versus 1578 days). Moreover, in the AAU patients, the younger age onset of AAU (less than 30 years old here) would lead to an earlier diagnosis of AS later with a median of 1445.5 (742–2241) versus 1544 (819–2289) days of survival for the group of age onset of AAU greater than 30 years old. The difference is statistically significant (*p* < 0.05). Conclusions: AAU was a risk factor for AS. To identify AAU as an extra-articular manifestation is crucial for early diagnosis and treatment of AS and containing functional loss accordingly.

## 1. Introduction

Ankylosing spondylitis (AS) is a chronic, inflammatory rheumatic disease, which is more popular in white Caucasian males; the incidence is estimated as three to seven per 100,000 person-years and prevalence estimated as up to 0.6% in Western populations [1]. The clinical presentations of AS might be insidious; it could take five to ten years for a confirmed diagnosis. AS usually attacks the sacroiliac joints and axial skeletons, which are the major targets that physicians are familiar with, but other organs such as inflammation of the uvea (so called uveitis) in the eyes especially acute anterior uveitis (AAU) is most often encountered [2]. The significance of extra-articular manifestations of AS has been highlighted in recent decades because these extra-articular manifestations (EAMs) could impact the prognosis of AS, increase the usage of healthcare resources, and affect the quality of these patients’ lives due to worse outcomes by EAMs. On the other hand, clinicians could build up diagnosis of AS earlier based on EAMs. Brewerton et al. first reported the association between HLA B27 and AAU in 1973 [3]. Carvalho et al. also found that 38 cases were with underlying spondyloarthritis disease groups in 100 non-granulomatous uveitis patients [4]. Uveitis is a rare disease; the incidence rate of uveitis is estimated as 17 and 52.4 cases per 100,000 people [5], and the prevalence between 38 and 370 per 100,000 populations [5,6,7,8,9]. Yet uveitis is the top fourth leading cause of blindness in developed countries [10]. Thus, the significance of uveitis cannot be over-emphasized. AAU is a form of acute-onset inflammation in the iris and or ciliary body (so-called iritis and/or iridocylitis). Granulomatous or non-granulomatous, the duration should be less than three months. Clinical presentations of AAU encompass pain, congestion, tearing, photophobia, and blurry vision, so AAU patients usually seek emergency care or healthcare as soon as possible. The etiology of uveitis might have idiopathic, immunologic, infectious, or traumatic origins [11]. Among the association with systemic diseases of immunologic origins, especially for AAU, AS is one of the most well-known systemic diseases. As the manifestations of AS often need multidisciplinary approaches which include ophthalmology, rheumatology, and orthopedic fields, it is crucial to build interdisciplinary collaboration for earlier diagnosis and initiation of treatment to deter and/or lessen later functional loss [12]. In clinical settings, the ophthalmologist could be the first physician to diagnose the patient and detect the associations based on AAU eye conditions. Therefore, the association between initial uveitis and later development of AS should always be considered by ophthalmologists. Functional loss usually develops in the first decade of AS, so the sooner the diagnosis is made, the better the outcome for the patient is likely to be. This study is aimed at delving into the incidence rate of AAU and the relative hazard ratio of AAU to develop AS later in Taiwan based on National Health Insurance Longitudinal Research Database.

In Taiwan, the government launched national health insurance as a mandate on 1 March 1995, and the coverage rate is around 99% [13,14]. A national wide population study using a longitudinal case-controlled cohort study was conducted to examine whether AAU was a risk factor for AS. The Longitudinal Health Insurance Database 2000 (LHID2000) is a sub-dataset of the National Health Insurance Research Database (NHIRD) and includes all claim data (from 1996 to 2008) of one million beneficiaries who were randomly selected from the system in 2000. There was no significant difference in age, sex, or average insured payroll-related premiums between the sample group and all enrollees.

## 2. Materials and Methods

### 2.1. Selection of Patients and Variables

This cohort comparison study consisted of all patients diagnosed with AAU (n = 5621) (ICD-9-CM codes 364.00) from ambulatory (including emergency) care and inpatient care, from 1 January 2000 to 31 December 2008 in the LHID 2000. For each case of AAU patient, four patients without AAU were matched to the control group, which were randomly selected from the dataset. The patients included in the study and the control group patients were matched by sex, age, and the index dates of ambulatory care visits (including outpatient clinic and emergency department) or hospitalization for the initial diagnosis of AAU patients. Among these datasets, we further examined all patients who had ever developed AS (ICD-9-CM codes 720.0). Demographic data, such as sex and age, were recorded. The inclusion criteria for this cohort comparison study were based on ICD-9-CM codes, which were entered by the practicing physicians. The AAU patients were divided into two groups based on ages ≤30 years old and ages >30 years old and then analyzed whether younger age onset of AAU (≤30 years old here) would lead to an earlier diagnosis of AS later by a paired Student’s *t*-test of their survival date.

This study was approved by the institutional review board of Taipei Medical University, Taiwan (JIRB-N201602088). Since this study analyzed de-identified data, the review board waived the requirement for written informed consent from the patients involved.

### 2.2. Statistical Analysis

SAS for windows 9.3 (SAS Institute, Inc., Cary, NC, USA) was used for this study. Descriptive statistical analyses were performed to compare the characteristics of the cohorts in terms of demographic characteristics, and the risk of developing AS. The risk of AS between AAU patients and controls was compared by estimating the crude hazard ratio with logistic regression. Logistic regression is widely used in analysis of categorical data, especially data with variables that have binary responses. It can predict a dichotomous outcome using independent variables. This dichotomous outcome is the presence or absence of AS in this study. Kaplan–Meier analysis was used to calculate the cumulative incidence rates of developing AS between the cohorts, and the log-rank test was used to analyze the differences between the survival curves. Thereafter, separate Cox proportional hazard regressions were performed to compute the AS-free rate after adjusting for possible confounding factors such as age and sex. Cox regression is a method for investigating the effect of variables upon the time a specified event takes to happen. The coefficients in a Cox regression relate to hazard; a positive coefficient indicates a worse prognosis, and a negative coefficient indicates a protective effect of the variable with which it is associated. Statistical significance was set at *p* ≤ 0.05.

## 3. Results

### Demographic Data

Between 2000 and 2008, 5621 AAU patients and 22,484 AAU control patients with age- and sex-matched controls were recruited, after excluding ineligible subjects. The median age of the AAU patients was 47.0 (interquartile range as 33.0–63.0) years old—for the controls it was 47.3 (interquartile range as 32.0–63.0) years old. The sex ratio in the cohort group was as follows: M/F 56.13%/43.87% in the AAU study group and 56.13%/43.87% in the AAU control group (Table 1).

For the AAU study group, there were 5621 patients, and, among them, 188 patients (3.34%) developed AS later; for the AAU control group, there were 22,484 patients, and 288 patients (1.28%) that developed AS later. Therefore, there were 476 AAU patients (1.69%) of 28,105 cases that developed AS of the AAU patients and AAU control group in total.

The differences in the risk of developing AS in the cohort group were statistically significant (*p* < 0.0001). The median observation period and interquartile range in the cohort group to develop the AS was 1507 (591–2423) days in the AAU study group, and 1578 (708–2448) days in the AAU control group. The differences in the survival analysis in the cohort group are statistically significant as well. The *p*-value is <0.0001 (Table 2). In the AAU patients, the younger age onset of AAU (less than 30 years old here) would lead to an earlier diagnosis of AS later with a median 1445.5 (742–2241) versus 1544 (819–2289) days of survival for the group of age onset of AAU greater than 30 years old. The difference is statistically significant (*p* < 0.05).

**Figure 1:**
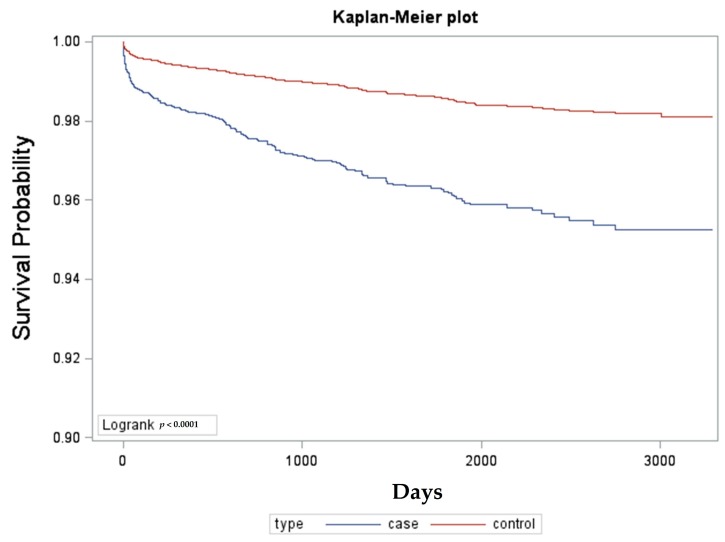
Kaplan–Meier Survival analysis of AAU patients against AS-free time.

Among the cohort, the crude hazard ratio by logistic regression with a 95% confidence interval for the AAU patients to develop AS is 2.667 (2.214–3.213). The adjusted hazard ratio by Cox proportional regression model is 2.705 (2.251–3.251). The adjusted factors included age and sex. Kaplan–Meier survival analysis was conducted to examine the cumulative incidence rates of developing AS between the cohort group, and a log-rank test was used to probe the differences between the survival curves. The results (Figure 1) revealed statistically significant differences between the cohort group (*p* < 0.0001). In AAU cases in which AS was developed, neither sex nor age increased the hazard ratios.

## 4. Discussion

The incidence rate of AAU in Taiwan was 0.56% in the present study, the median age and interquartile range were 47.0 and 33–63 years old, respectively, with slight male preponderance, and the gender ratio of male to female was 1.28 (M/F 56.13%/43.87%). The annual incidence of acute anterior uveitis was estimated as 0.012% to 0.016% in the Northern Finland and resident Rochester population [15]; among them, 50%–90% was reported as anterior uveitis. Gender ratios (male/female) of AAU have been reported from 1.1 to 2.5; in Monnet’s study, the M/F ratio is 1.3, which is very close to ours (1.28) [16]. The median age of our AAU study group is 47 years old, which is much older from other studies of uveitis or anterior uveitis, as the mean or median age of the onset age is around 31 to 38 years old [12,17,18]. The reason for the discrepancy might be because the inclusion criteria of our study is by the first visit of ICD-9-CM coding from 2000 to 2008 in the longitudinal NHIRD. However, in Llorec’s four-year longitudinal cohort uveitis study in Spain’s tertiary hospital, the median age was 45 years, similar to our study, except that our sampling is with respect to AAU instead of uveitis. The prevalence or incidence rate on previous reports might have biases for a higher proportion of intermediary, posterior, and pan-uveitis, as they were based on tertiary-referred centers mostly, and ours is based on the incidence rate of AAU and on our longitudinal NHIRD and thus is a nationwide population-based study. However, there is no audit towards the physician’s coding, so accuracy cannot be warranted. The epidemiology of uveitis is diverse, from different ethnic and geographical patterns [18], whereas our study is representative of an Asian population and cannot be generalized in other ethnicities or globally.

The annual incidence rate of AAU in HLA-B27-positive families without AS was estimated at 2% and 10% in HLA-B27-positive AS families and patients. In another population study in Netherlands, AAU prevalence in the general population is approximately 0.1% and 1% in the HLA-B27 positive population [19]. Thanks to the advancement of molecular biology and the genomic era, the diversity of AS presentations has been proposed due to HLA-B27 subtypes, as HLA-B27 positivity is highly correlated to AS, even as high as 90% of cases in some studies [20]. In studies of patients of Northern European descent, HLA-B*2705 is the most frequently associated with AS and related spondyloarthropathies, which include AAU. On the contrary, in Asian descendents, which includes Japanese and Chinese, HLA-B*2704 is more predominant, and the association with AS and AAU is not as high as that compared with HLA-B*2705. Other subtypes HLA-B*2702 and HLA-B*2703 are much less frequent and are prevalent in other areas. In our study, we recruited study samples by ICD-9-CM coding based on NHIRD and cannot verify whether their HLA-B27 is positive or negative, which is one of our study’s limitations. However, we do believe that this association still exists.

The results showed AAU is associated with increasing the risk of developing AS later, as the crude hazard ratios with a 95% confidence interval were 2.667 (2.214–3.213); and the adjusted hazard ratios with 95% confidence interval were 2.705 (2.251–3.251) for AAU patients. This means that the hazard ratios, regardless of whether the crude or adjusted ones were adjusted for age and sex, are relatively high for AAU patients for developing AS later. This result verified our previous hypothesis, as AAU is a risk factor in developing AS later. Most studies addressing associations between AS and AAU have been conducted on extra-articular manifestation involvement, which encompasses AAU, psoriasis, and inflammatory bowel disease. In a study by Stolwijk et al. [12], patients with AS had a 16-fold increase in hazard ratios for developing AAU later and most often occurred after the first year of diagnosis. However, the time frame in our study is different; we recruited AAU subjects first and investigated their hazard ratio for developing AS later after nine years, and our hazard ratio is around 2.7-fold higher than the control group in this case-controlled cohort study. The reason for this discrepancy might be the different time frame, different ethnicities, and different approaches of methodology such as the stratification of subjects by age ranks in Stilwijk’s study [12]. Another between-group AAU analysis based on groups aged less than or equal to 30 years old and greater than 30 years old was performed and revealed that the younger age group with AAU corresponded with an earlier diagnosis of AS by survival day analysis, and the difference is statistically significant. This result denoted that our research subjects might comprise AAU with varied etiology and implicated the possibility of under-estimation of HLA-B27 prevalence because we used a claims database, and this is indeed a flaw in this study and also a limitation of this study.

We also found that, between the two groups, it took around four years (1507 (591–2423) days) for the AAU patients to develop AS; on the contrary, it took 1578 (708–2448) days for the control group to develop AS later. Thus, our data suggests that ophthalmologists could be the first physicians to help diagnose AS patients with extra-articular manifestations, which is AAU here. We conducted this investigation to raise awareness of the association between ocular co-morbidities (AAU) and systemic diseases (AS). We suggest that HLA-B27 should be a part of studies of patients with AAU. In patients with AAU, careful evaluation and follow-up are critical, including the recognition of non-radiographic AS. On the other hand, rheumatologists or internists handling AS should be familiar with the possibilities of developing AAU as well. The reports [20] revealed that male AS patients usually start with sacroiliac joint and axial skeleton involvement first and develop AAU (extra-articular manifestation) later; on the other hand, the counterpart female AS patients start with AAU (extra-articular manifestation) first and usually develop atypical arthritis (which means peripheral joints other than sacroiliac or axial skeleton joints) later. In this way, the ophthalmologists ought to take more precautions serving first-onset AAU female patients and refer the patients to rheumatologists, as this could lead to earlier diagnoses of AS.

This study was conducted based on a single-payer, longitudinal, national health insurance database in Taiwan to probe the hazard ratios of AAU as risk factors for AS development. Were it not for this NHIRD longitudinal dataset, it would be very time-consuming and costly to collect enough cases to conduct research, especially since the case number of AAU is so low; AAU could even be defined as a rare disease based on the epidemiology implications. Moreover, since it is a national health insurance database, it is a population-based study, so the results are more robust and convincing without selection bias.

On the other hand, the claims data of the database does not show important clinical features such as the HLA-B27 test results, radiological image results, and skin and/or bowel manifestations. Nor does it show personal health records, such as previous bowel infections or important life events related to stress, which are important in triggering AS. There are also flaws in the claims data—for example, our inclusion criteria were based on ICD-9-CM codes, and there is no way to verify the accuracy of coding by physicians. Thus, there are some expected defects in this respect using a national health insurance research database.

## 5. Conclusions

Taiwan’s national health insurance database was used to examine the association of AAU with the later development of AS. The results revealed that AAU increases the risk of later-developing AS, as the crude HR with a 95% confidence interval was 2.667 (2.214–3.213), and the adjusted HR with a 95% confidence interval was 2.705 (2.251–3.251). AAU occurred before AS in some cases, presenting around four years earlier in clinical presentation—the AS-survival-free days and interquartile range for AS were 1507 and 591–2423 days, respectively. Further, in the AAU patients, the younger age group with onset of AAU (less than 30 years old here) led to an earlier diagnosis of AS later with a median of 1445.5 (742–2241) versus 1544 (819–2289) days of survival for the age group with onset of AAU greater than 30 years old. The difference is statistically significant (*p* < 0.05). We suggest that ophthalmologists should detect underlying causes of AAU to make early diagnosis and treatment of AS accordingly so that disease can be contained and possible functional loss or disabilities, which include not only articular involvement but also extra-articular involvements, can be prevented.

## Figures and Tables

**Table 1:** Demographics of AAU group and control group.

Variable	AAU Patients (n = 5621)		Control Group (n = 22,484)		*p*-Value
	n	%	n	%	
Age, median (IQR ^a^)	47 (33–63)		47.3 (32–63)		0.99
Gender					0.99
Male	3155	56.13	12,620	56.13	1.0
Female	2466	43.87	9864	43.87	1.0
AS	188	3.34	288	1.28	<0.0001 ***
Observation time without developing AS (days, median, IQR ^a^)	1507 (591–2423)		1578 (708–2448)		<0.001 ***

^a^ IQR: Interquartile range, *** indicated *p* < 0.001.

**Table 2:** Crude and adjusted hazard ratios for developing AS among patients with AAU and the control group during the ten-year follow-up (n = 28,105).

Development of AS	Total		Patients with AAU		Control Group	
	No.	%	No.	%	No.	%
Nine-year Follow-up Period						
Yes	476	1.69	188	3.34	288	1.28
No	28,105	98.72	5621	96.66	22,484	98.72
Crude HR (95% CI)	-		2.667 (2.214–3.213)		1.00	
Adjusted ^a^ HR (95% CI)	-		2.705 (2.251–3.251)		1.00	

^a^ Adjustments were made for sex and age. 95% CI: 95% confidence interval.
